# Understanding the factors that effect maximal fat oxidation

**DOI:** 10.1186/s12970-018-0207-1

**Published:** 2018-01-12

**Authors:** Troy Purdom, Len Kravitz, Karol Dokladny, Christine Mermier

**Affiliations:** 1 Department of Health, Athletic Training, Recreation, and Kinesiology, Longwood University, 201 High St, Farmville, VA 23909 USA; 2 Department of Health, Exercise & Sports Sciences, University of New Mexico, Albuquerque, NM USA; 3 Department of Gastroenterology, The University of New Mexico, Albuquerque, NM USA

**Keywords:** Fat oxidation, Substrate oxidation, Dietary fat oxidation, Crossover concept, Maximal fat oxidation, PDH activity, Fat adaptation, Ketogenic diet, Cpt-1, Carnitine

## Abstract

Lipids as a fuel source for energy supply during submaximal exercise originate from subcutaneous adipose tissue derived fatty acids (FA), intramuscular triacylglycerides (IMTG), cholesterol and dietary fat. These sources of fat contribute to fatty acid oxidation (FAox) in various ways. The regulation and utilization of FAs in a maximal capacity occur primarily at exercise intensities between 45 and 65% VO_2max_, is known as maximal fat oxidation (MFO), and is measured in g/min. Fatty acid oxidation occurs during submaximal exercise intensities, but is also complimentary to carbohydrate oxidation (CHOox). Due to limitations within FA transport across the cell and mitochondrial membranes, FAox is limited at higher exercise intensities. The point at which FAox reaches maximum and begins to decline is referred to as the crossover point. Exercise intensities that exceed the crossover point (~65% VO_2max_) utilize CHO as the predominant fuel source for energy supply. Training status, exercise intensity, exercise duration, sex differences, and nutrition have all been shown to affect cellular expression responsible for FAox rate. Each stimulus affects the process of FAox differently, resulting in specific adaptions that influence endurance exercise performance. Endurance training, specifically long duration (>2 h) facilitate adaptations that alter both the origin of FAs and FAox rate. Additionally, the influence of sex and nutrition on FAox are discussed. Finally, the role of FAox in the improvement of performance during endurance training is discussed.

## Background

Lipids are the substrate largely responsible for energy supply during submaximal exercise [1–3]. However, the definitive role of lipid contribution during cellular respiration has yet to be fully elucidated. Subcutaneous adipose tissue, intramuscular triacylglycerides (IMTG), cholesterol, and dietary fat all contribute to fatty acid oxidation (FAox) [1]. Moreover, the energy contribution from lipid oxidation during submaximal exercise is in addition to carbohydrate oxidation (CHOox) [4]. However, as exercise intensity increases, the contribution of carbohydrate oxidation increases in proportion to the decrease in lipid oxidation [4]. Nonetheless, the oxidation of lipids is the predominant fuel source (%) during submaximal exercise intensities (<65%VO_2max_) [1, 2, 5]. Increases in exercise intensity that exceed 65% of VO_2max_ produces a shift in energy contribution favoring CHOox. A term used to describe the point when lipid oxidation reaches maximum is maximal fat oxidation (MFO). Exercise intensities that exceed MFO oxidize CHO in greater proportion [2, 4, 5].

Maximal fat oxidation has been reported to occur between 47 and 75% of VO_2max_, and varies between trained and untrained men and women [1, 5, 6]. Nonetheless, MFO has been observed to range from 0.17–1.27 g/min [7], where ketogenic adapted individuals can exceed ≥1.5 g/min [3]. Factors that alter lipid oxidation rates are training status, exercise intensity, duration, sex, and nutritional intake [1]. Each of these factors facilitate or inhibit physiological changes that influence FAox [1] and are discussed in subsequent sections.

## Lipid oxidation

### Lipolysis

Triacylglycerol (TAG) is the stored form of fat found in adipocytes and striated muscle, which consists of a glycerol molecule (a three-carbon molecule) that is bound to three fatty acid (FA) chains. Fatty acid chains are carbon molecules linked together with accompanying hydrogen atoms. The intercellular process of liberating the FAs from the glycerol backbone is called lipolysis [8–10]. Once this occurs, FAs are released into the blood and transported to working muscle for oxidation.

Adipose tissue reserves can store a significant amount of TAG and deliver a seemingly endless supply of energy for prolonged exercise performance. A person with 7–14% body fat has >30,000 kcal of energy reserves stored in adipose tissue [3]. Therefore, if exercise intensity is maintained below 65% VO_2max_, exercise can theoretically be mainted for longer durations because of the oxidation of endogenous TAG stores. However, when exercise intensities exceed ~65% VO_2max_, FAox is reduced increasing the reliance on CHO for energy [2, 4, 11].

The process of lipolysis is largely controlled via the endocrine system [12]. The release of epinephrine stimulates lipolysis and therefore increases serum FA concentrations. At rest, catecholamine (epinephrine) concentrations in the blood are low. As exercise intensity increases, there is a simultaneous and progressive increase in epinephrine [13] from the adrenal glands. Depending on exercise intensity and/or duration, catecholamine concentrations can increase >20 times above basal levels [14]. The exercise-induced catecholamine release stimulates lipolysis, liberating FAs from the glycerol molecule [8, 15]. During exercise intensities equating to ~60% VO_2max_, serum FA concentrations increase 2–3 times resting values [16].

The binding of epinephrine to the *β*-adrenergic receptor on adipose cell membranes triggers a cascade of events that begin with the phosphorylation of adipose triglyceride lipase (ATGL) [8, 9]. Recent findings indicate that lipolysis is under a hierarchal regulation by ATGL and hormone sensitive lipase (HSL) [8, 9, 17]. Additionally, studies have shown that ATGL has a greater sensitivity to epinephrine (a 10-fold increase) compared with HSL [8]. Therefore, ATGL disassociates the first FA from the glycerol molecule forming diacylglycerol + FA or (DAG), whereas HSL is responsible for the second FA chain disassociation [8]. Lastly, the catabolism of the monoacylglycerol is facilitated by monoglycerol lipase where the FA is transported and the glycerol is utilized in glycolytic or gluconeogenic pathways, mostly in the liver [10].

Endogenous skeletal muscle FAs, termed IMTGs, may contribute to overall FAox independent of serum FA contribution [18, 19]. Intramuscular triacylglyerides are arranged within striated muscle, primarily in type I fibers in close proximity to the mitochondria [19, 20]. The process of liberating intramuscular FAs from the TAG molecule for oxidation is slightly different from peripheral adipose tissue. Transport across the cell membrane is not a limitation to IMTG oxidation due to the fact that they are stored within the muscle cell. However, the lipolytic enzymes lipoprotein lipase (LPL) and HSL are necessary to mobilize FAs (lipolysis) from the intracellular glycerol molecule [9]. Lipoprotein lipases are lipoproteins bound to the intramuscular capillary endothelium, and responsible for liberating the first FA from the TAG molecule within the cell, forming DAG [21].

The process of oxidizing IMTGs is facilitated by HSL and is similar to subcutaneous adipose tissue derived HSL. Hormone sensitive lipase has three important characteristics that impact DAG oxidation. First, HSL demonstrates a 10-fold higher affinity to DAG compared to TAG [20]. Secondly, HSL operates optimally at a pH of 7.0 and activity is increased as exercise intensity rises [20]. Lastly, HSL is directly stimulated by epinephrine and independent of the energy sensitive cAMP cascade known to stimulate lipolysis [18, 20].

Despite the known presence of IMTG within muscle (primarily with endurance trained subjects and type II diabetic subjects) [20], the overall IMTG concentration and energy contribution is still under debate due to tissue variabilities [9, 18, 22]. Some of the speculation is that ~10% of serum derived FAs are used to replenish IMTGs during exercise [13]. This makes it difficult to quantify the actual contribution of IMTGs to exercise substrate demands. Additionally, variation in methodologies, e.g. muscle biopsy, isotope tracers, magnetic resonance spectroscopy make comparative efforts challenging [23]. Lastly, disparity in training status and dietary macronutrient specificity further complicate the ability to obtain definitive conclusions. More research in the area of IMTG energy flux is necessary to determine IMTG influence on energy contribution during exercise.

### Fatty acid transport

Limitations to FAox are due in part to a multi-faceted delivery system that has a series of regulatory events [18]. Once FAs leave the adipocyte they first bind to albumin, which can bind as many as 12 FA molecules [15]. Interestingly, due to poor circulation in peripheral adipose tissue and an increased ratio of FA:albumin after exercise, the albumin binding capacity may be surpassed and high levels of unbound serum free fatty acids can create a harmful condition [15]. Due to poor circulation in type II diabetics, a high percentage of liberated FAs as a result of exercise-induced, catecholamine-stimulated lipolysis are not released into the circulation during high intensity exercise [13]. However, endurance training has been shown to increase blood flow to subcutaneous adipose tissue by 2–3 fold [13], which can increase overall FA transport to working muscle. Despite the positive circulatory effects of endurance training, limitations to the rate of FAox appear to be mediated by cellular transport rather than systematic transport of serum FAs from adipose tissue [24].

Fatty acid transport across the muscle cell membrane occurs via transport proteins, mainly CD36 [24, 25]. CD36 appears within the plasma membrane in as little as 1 min after the initiation of muscle contraction [25]. Schenk and Horowitz (2006) [26] reported that sedentary obese women training at >70%HRmax increased CD36 expression by 25%. The result of regular endurance exercise and the corresponding increase in CD36 within muscle cell membranes is highly correlated (R^2^ = 0.857, *P* < 0.003) with a 23% increase in resting FAox [26]. Moreover, CD36 upregulation occurs rapidly and remains elevated for 3 days post exercise. Schenk and Horowitz (2006) [26] showed that the plasticity of the cellular changes due to endurance training positively influence resting FAox (23%) for days after exercise concludes.

In humans, sex differences have been shown to effect CD36 expression [27, 28] due to circulating estrogen concentrations [29]. After 90 min of cycling at 60% VO_2max_, CD36 mRNA was 85% higher in women vs men. Interestingly, there is a 49% greater FA uptake ability due to greater CD36 protein concentrations in trained women compared to trained men [30]. Additionally, Kiens et al. 2004 [30] state that CD36 protein concentrations are 49% higher in women compared to men, irrespective of training status.

In summary, transport of FAs across the cell membrane positively affects FAox [13, 26, 30]. Endurance training increases CD36, thereby increasing intracellular transport for oxidation. Increasing transport of FAs into the cell for oxidation spares CHO stores for both high intensity exercise and prolonged exercise [11].

### Within-cell FA transport into mitochondrion

Within the cell, FA chain type and length have been shown to determine oxidative rates within the mitochondrion largely due to transport specificity [31]. An inverse relationship of FA carbon chain length and oxidation exists where the longer the FA chain the slower the oxidation [31]. Interestingly, this relationship inspired the supplementation of short and medium chain fatty acids (MCFA) as an ergogenic aid. However, while significant increases in FAox were observed with MCFAs compared to LCFAs [32], no differences were observed in endurance performance [32, 33]. Jeukendrup and Aldred [33] suggest this may be due to the transport and rapid oxidation of MCFAs independent of carnitine palmitoyltransferases. Intuitively, this would seem advantageous, however the rapid transport and oxidation of short and MCFAs is suspected to increase ketone production opposed to increased exercise performance [33]. Ketones are a viable fuel source recognized largely as a positive ketogenic diet adaptation [34], however, high intensity exercise relies primarily on glycolytic metabolism for ATP supply and therefore may be compromised [35]. This concept is discussed in detail in subsequent sections.

**Fig. 1:**
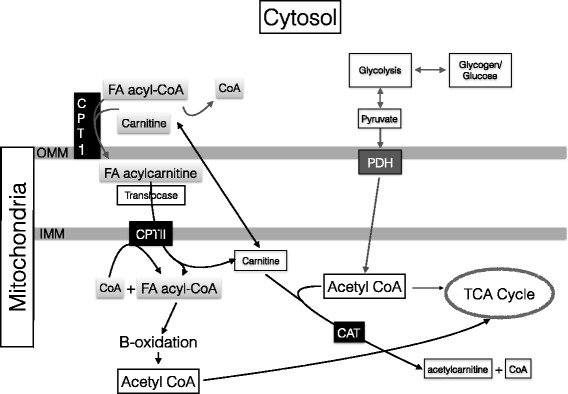
Proposed interaction within skeletal muscle between fatty acid metabolism and glycolysis during high intensity exercise. During high intensity exercise the high glycolytic rate will produce high amounts of acetyl CoA which will exceed the rate of the TCA cycle. Free carnitine acts as an acceptor of the glycolysis derived acetyl groups forming acetylcarnitine, mediated by carnitine acyltransferase (CAT). Due to the reduced carnitine, the substrate for CPT-1 forming FA acylcarnitine will be reduced limiting FA transport into the mitochondrial matrix. This limits B-oxidation potential reducing overall FAox. OMM: outer mitochondrial membrane; IMM: inner mitochondrial membrane; CPT-1: carnitine pamitoyltransferase; FA: fatty acid; CPT-II: carnitine palmitoyltransferase II; PDH: pyruvate dehydrogenase; CAT: carnitine acyltransferase. Adapted from Jeppesen and Kiens 2012

The slowed oxidation of serum derived and IMTG long chain FAs (LCFAs) (>12 carbons) are due to the requirement of a mitochondrial transport protein for LCFA transport [36]. The transport protein known as carnitine palmitoyltransferase-1 (CPT-1) is located on the outer mitochondrial membrane and is responsible for the transportation of LCFAs into the mitochondria shown in Fig. 1 [35, 37, 38]. Fatty acids with 12 or fewer carbons are classified as short or MCFAs and can pass through the mitochondrial membrane independent of protein transporters [31, 33, 38]. Nonetheless, CPT-1 is necessary for LCFA transport, a product of free carnitine, and is found in both the cytosol and mitochondrial matrix shown in Fig. 1 [37, 38].

CPT-1 concentration, located within the mitochondrial membrane during exercise appears to be regulated in part by exercise intensity [24, 38]. No significant changes in CPT-1 concentrations were observed in subjects exercising at lower exercise intensities (50% VO_2max_) compared to rest [24]. However, exercising at 60% VO_2max_ has been shown to increase CPT-1 concentrations. At exercise intensities >75% VO_2max_, muscle free carnitine concentrations decrease progressively [24] and therefore CPT-1 can be a FA transport limitation, ultimately reducing FAox at higher exercise intensities [24, 38].

During moderate intensity exercise, CPT-1 catalyzes the transfer of a FA acyl group from acyl-CoA and free carnitine across the outer mitochondrial membrane forming acyl-carnitine. Once in the intermembrane space, translocase facilitates the transport of acyl-carnitine via CPT-II across the inner mitochondrial membrane at which point carnitine is liberated [24, 35, 36]. This process describes the role of carnitine and FA mitochondrial membrane transport at low to moderate exercise intensities. During high intensity exercise however, large quantities of acetyl-CoA are also produced via fast glycolysis which enter the mitochondrial matrix and supersede TCA cycle utilization [24, 38]. The result of the abundant glycolytic derived acetyl-CoA forms acetyl-carnitine and monopolizes the available free carnitine limiting FA derived acyl-CoA transport. Thus, free carnitine is used to buffer excess glycolytic derived acetyl-CoA by forming acetyl-carnitine [24, 35, 38], and therefore the limited concentration of free carnitine is a rate limiting step in FA transport/oxidation (Fig. 1).

Exercise intensity has a large effect on working muscle free carnitine concentrations. Compared with resting conditions, exercising at intensities greater than 75% VO_2max_ have been shown to reduce free carnitine concentrations in working muscle by ~80% [37]. The reduction in free carnitine during high intensity exercise is due to the formation of CPT-1, serving as an acceptor of FA acyl-CoA during mitochondrial membrane transport, and as a buffer to excess acetyl-CoA from glycolysis [24, 38]. Therefore, as exercise intensity increases beyond moderate intensity, carnitine can be a limitation of FA substrate utilization due to the buffering of glycolytic acetyl-carnitine during high intensity exercise [24, 37, 38]. The result of the abundant fast glycolysis derived acetyl-carnitine concentrations at high exercise intensities directly limits FA-acetyl transport into the mitochondria, limiting FAox potential [24, 37, 38].

### Fatty acid oxidation

Fatty acid oxidation or beta-oxidation (beta-ox) describes the catabolic process of removing H^+^ ions from FAs while producing acetyl-CoA, which is further metabolized within the TCA cycle. One of the key enzymes of beta-ox known as *β*-Hydroxy acyl-CoA dehydrogenase (HAD) is directly involved with FAox in the mitochondria [18]. Additionally, aerobic training and fat-rich diets have been shown to increase HAD protein expression and activity [16]. Fatty acid oxidation is directly influenced by HAD activity [1, 18] in addition to the transport of FAs across the cellular and mitochondrial membranes [24, 37, 38].

While FAox fluctuates continuously, the endocrine system is principally responsible for the regulation of lipid oxidation at rest and during exercise [15]. The hormonal mechanisms that stimulate lipid metabolism are based primarily on catecholamines [12], cortisol, growth hormone, where insulin is inhibitory [16]. Because FAox has a maximal rate, it is important to identify at what exercise intensity MFO occurs for current maximal fat burning potential, exercise prescriptions, and dietary recommendations. Identifying the stimuli that influence fat oxidation is necessary to best give exercise recommendations for the exercise intensity that facilitates optimal fat burning potential.

## Factors that influence maximal fat oxidation

### Training status

Maintaining an elevated training status impacts FAox potential due to the increase in IMTG, cellular/mitochondrial protein changes, and hormonal regulation. The adaptations that occur due to regular endurance training favor the ability to oxidize fat at higher workloads in addition to increasing over all MFO [39, 40]. Increased fat oxidation has been shown to improve with endurance training, and therefore increases in MFO parallels changes in training status. Bircher and Knechtle, (2004) [41] demonstrated this concept by comparing sedentary obese subjects with athletes and found that MFO was highly correlated with respiratory capacity, and thus training status.

Trained subjects possess a greater ability to oxidize fat at higher exercise intensities and therefore demonstrates the correlation between respiratory capacity and MFO [27, 41, 42]. However, a similar rate of appearance in serum glycerol concentrations is observed in sedentary vs. trained subjects [27]. These results, however, conflict with results from Lanzi et al. (2014) [43] who reported that obese subjects had a higher serum FA rate of appearance likely due to increased total adipose tissue mass (kg). Furthermore, sedentary/obese subjects have a reduced cellular transport and fat oxidation capabilities, therefore maintaining higher serum FA concentrations [43]. Despite the reported reduced rate of glycerol appearance for the trained population reported by Lanzie et al., trained women were shown to oxidize fat at twice the rate compared with the obese population [41].

The training effect, and therefore an increase in respiratory capacity is partially the result of an increase in MFO. Scharhag-Rosenberger et al. (2010) [40] conducted a prospective study to demonstrate this concept using sedentary subjects who met or exceeded ACSM’s minimum cardiorespiratory exercise recommendations for a period of 1 yr. Maximal fat oxidation (rate) increased over 12 months of training (pre-training 0.26± 0.10; post-training 0.33± 0.12 g/min) and it occurred at a higher exercise intensity (pre-training 35±6% VO_2max_; post-training 50±14% VO_2max_). The training status effect on MFO further applies to athletic populations. In moderate vs highly trained subjects, the exercise intensity (%VO_2max_) that MFO occurred was not significantly different, but MFO was elevated for the highly trained subjects (0.29±10 vs 0.47±.17 g/min, respectively) [39]. Furthermore, mitochondrial enzymes citrate synthase and HAD were found to be significantly increased (49% and 35%) in highly trained vs. moderately trained participants respectively [42]. Increasing HAD directly elevates beta-ox rate while citrate synthase increases the TCA cycle rate [44]. This evidence suggests that lipolysis and systemic FA delivery are not limitations to FAox at higher exercise intensities. Therefore, FA cellular transport proteins (CD36 and CPT-1) [24, 25] and mitochondrial density (HAD) are likely the limitation of FAox during high intensity exercise [42]. Elevating FAox potential by increasing cellular respiration capacity increases FAox at higher exercise intensities which can have a positive influence on aerobic capacity.

Acknowledging the occurrence of large inter-individual differences in MFO, differences in MFO relative to training status are still observed [39]. Lima-Silva et al. (2010) [39] showed that differences in the lipid oxidative potential may exist in high vs. moderately trained runners referenced above. However, while no statistical differences were observed between groups at the exercise intensity that MFO occurred, there was an increased capacity to oxidize fat in the highly trained subjects. It is worth noting that the increased performance capacity in highly trained runners is most likely attributed to an increased CHO oxidative potential at higher exercise intensities in order to maintain higher steady state running workloads [39]. Subsequently, cellular protein expression, oxidative capacity and therefore training status do have the ability to influence fat oxidation.

Training status further influences maximal fat oxidative potential by increasing endogenous substrate concentrations [19, 20]. Endurance training enhances type I fiber IMTG concentrations as much as three-fold compared with type II fibers. Increased MFO potential due to endurance training is further influenced by IMTG FA-liberating HSL [22] and LPL proteins [20], which are responsible for the liberation of intramuscular FAs from the IMTG molecule. However, during exercise, the IMTG pool is constantly being replenished with plasma-derived FAs during exercise [20, 45]. Nonetheless, the reliance of IMTGs during submaximal exercise durations lasting <2 h is essential to maintaining workloads [45]. The exercise duration effect could be due to *β*-adrenergic receptor saturation, which has been shown to occur during prolonged bouts of exercise [16, 46]. Furthermore, HSL activity has been shown to increase initially within 10-60 min, but returned to resting levels after 120 min of exercise, increasing reliance on serum derived FAs [20, 45]. More research in the area of hormone related FA kinetic limitations is warranted.

### Intensity

The exercise intensity that MFO occurs has been reported to range from 45–75% VO_2max_ [1, 4, 6, 41, 43], with a recent paper highlighting MFO rates from 1121 athletes in various disciplines (American Football, triathlon, golf, soccer, motor sports, cross county, and water sports amongst others) ranging from 23 to 89%VO2max [39]. Factors such as training status, sex, and nutrition [1] all impact FAox kinetics and thereore the exercise intensity that MFO occurs. Exercise intensity has the most profound effect on MFO based on a combination of events which include FA transport changes [24, 25] and hormone fluctuation, which can increase lipolytic rate [7]. The cellular and hormonal changes that occur during exercise are directly related to exercise intensity which can influence FAox [47].

Fatty acid oxidation varies relevant to exercise intensity and therefore examining lipid oxidation at specific exercise intensities is warranted. At 25% VO_2max_, FAox comprises >90% of energy expenditure and more specifically plasma FAs provide the largest energy contribution, where muscle glycogen and IMTG contribute very little [48]. At exercise intensities <65% VO_2max_ muscle glycogen and IMTG oxidation increase considerably to as much as 50% of energy expenditure, depending on exercise duration [15, 48]. Bergomaster et al. (2008) [49] compared 6wks of sprint interval training (Wingate Tests) to endurance training (~65% VO_2max_) and found no differences in MFO. These findings suggest that training ≥65% VO_2max_ will not increase MFO potential, which is in disagreement with more recent literature [39]. Previous research suggests that training at higher exercise intensities greatly influences substrate utilization [5, 42, 50]. It is worth noting that Bergomaster et al. [49] used moderately trained subjects (VO2max = 41.0 ± 2.0 ml/kg/min) where Achten et al. (2004) [5] and Nordby et al. (2005) [36] used higher trained subjects (VO2max = 58.4 ± 1.8 and 56.6 ± 1.3 respectively) to formulate their conclusions.

The increased expression of FAox transport and oxidative cell proteins (CD36, CPT-1, HAD, etc.) that results in an increase FAox are a result of exercise intensity [24, 49]. Bergomaster et al. (2008) [49] suggests a minimum training volume of two weeks is necessary independent of training status for sufficient cellular adaptation to occur. The Lima-Silva et al. (2010) [39] data, however, show that a heterogeneous sample of highly vs moderately trained subjects (VO_2max_ of 68.4 ± 4.5; 58.6 ± 5.4 ml/kg/min respectively) training for a minimum of 3 yrs. at varying exercise intensities had a 62% difference in fat oxidation rates (0.47 ± 0.17; 0.29 ± 0.10 g/min respectively). Thus, FAox adaptation potential is related to training at higher exercise intensities rather than non-descript chronic exercise adaptation. Additionally, it has also been shown that carnitine concentrations are a direct limitation of FAox (Fig. 1) at higher exercise intensities (>65% VO_2max_) to both IMTG [24] and serum FAox [38], regardless of mitochondrial enzymatic activity in untrained and moderately trained subjects. Interestingly, efforts to mitigate the limitations of free carnitine on MFO at high exercise intensities have been unsuccessful [24]. While exogenous carnitine supplementation increased muscle carnitine by 21% after 4 weeks of supplementation, no differences in performance were observed. While FAox was not measured, increases in muscle carnitine were able to buffer excess acetyl CoA by forming acetylcarnitine and thus increase pyruvate dehydrogenase (PDH) activity (38%) at 80% VO2max [24].

**Fig. 2:**
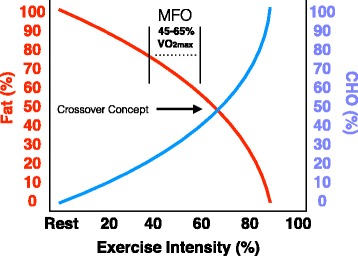
The crossover concept. The relative decrease in energy derived from lipid (fat) as exercise intensity increases with a corresponding increase in carbohydrate (CHO). The crossover point describes when the CHO contribution to substrate oxidation supersedes that of fat. MFO: maximal fat oxidation. Adapted from Brooks and Mercier, 1994

Exercise intensity may further influence MFO by influencing catecholamine concentrations which have regulatory effects on lipolysis [16], glycogenolysis, as well as gluconeogenesis [12]. Increased epinephrine concentrations that parallel increases in exercise intensity stimulate both glycogenolysis and gluconeogenesis [12]. As exercise intensity increases, so does catecholamine concentrations facilitating a concurrent increase of serum CHO and FAs into the blood [12]. However, the body still favors FAox at exercise intensities <65% VO_2max_ [5, 17]. When exercise intensity exceeds MFO, FAox (g/min) begins to decline; this process is described as the crossover concept [4] shown in Fig. 2.

The concept of the crossover point represents a theoretical means to understand the effect of exercise intensity on the balance of CHO and FA oxidation [4] (Fig. 2). More specifically, the crossover concept describes the point that exercise intensity influences when the CHO contribution relevant to energy demand exceeds FAox. The limitations of FAox at higher intensities is due to the vast amount of acetyl-CoA produced by fast glycolysis [24, 38]. The abrupt increase in total acetyl-CoA production at high intensity is due to fast glycolysis flooding the cell with potential energy, which suppresses FA mitochondrial transport potential resulting in decreased FAox (Fig. 1). Notably, the large inter-individual fluctuation of when the crossover point occurs at a given exercise intensity can be attributed in part to training status [39, 40]. Training status has been shown to effect catecholamine release and receptor sensitivity [12], endogenous substrate concentrations, and cellular transport protein expression; all of which contribute to the variability of when MFO occurs relevant to exercise intensity [1]. Nonetheless, MFO occurs in all populations regardless of training status, nutritional influence, etc., and is decidedly dictated in large part by exercise intensity [5, 6, 42].

### Duration

Another factor that significantly influences FAox is the duration of exercise [13, 45, 48]. Throughout a prolonged exercise bout, changes in hormonal and endogenous substrate concentrations trigger systematic changes in substrate oxidation [20, 51]. Studies show that endurance training promotes reliance on endogenous fuel sources for up to 120 min of submaximal exercise [47, 51, 52].

Exercise duration has a large effect on the origin of FAs for oxidative purposes. While the initiation of exercise relies heavily on endogenous fuel sources (IMTG and glycogen), reductions in IMTG concentrations have been shown to occur when exercise duration exceeds 90 min [45]. Beyond 90 min of submaximal exercise (≥65% VO_2max_), IMTG oxidation is mitigated by the increase in serum derived LCFAs [20, 45]. Increases in both epinephrine and plasma LCFA concentrations were observed when exercise exceeded 90 min with a simultaneous reduction in HSL activity. Therefore the increase in serum LCFAs [20, 45] and the saturation of HSL to epinephrine [16, 46] are postulated to inhibit HSL reducing IMTG oxidation when exercise exceeds 90 min [20]. When exercise exceeded 120 min of exercise, IMTG oxidation returned to resting values and was offset by a 46% increase [51] in serum FA delivery and oxidation [45]. Additional evidence shows that after 12 h of prolonged exercise, IMTG stores are 50–80% of pre-exercise concentrations despite the extreme duration of exercise [13].

The shift from intramuscular fuel sources to serum derived FAs after 2 h of submaximal exercise parallel changes in blood glucose concentrations. Untrained subjects who completed 3 hrs of knee extensions at 60% of 1RM had a 66% increase in serum glucose concentrations during the second to the third hour of exercise [51]. Trained subjects however experienced a reduction in muscular CHO uptake during the same time frame compared with the untrained. This suggests that the trained subjects were able to maintain FAox (despite substrate origin) during prolonged exercise to stave off CHO usage for high intensity exercise [51]. While the exercise intervention used in this study is not typically classified as endurance exercise, the exercise protocol does clarify the variation in the origin of substrate oxidation over time, and expands on the diverse effects exercise duration has on substrate oxidation.

Training duration has a large influence on FA and CHO oxidation during prolonged submaximal exercise. However, training status has little influence on the origin of FAs during the first 120 min of submaximal exercise. Nonetheless, trained subjects are able to maintain higher workloads with decreased metabolic work (HR) for longer periods compared to untrained individuals based on the ability to maintain FAox for longer durations [45]. Despite the training status effect on FAox, exercise duration will dictate substrate origin during submaximal exercise [20, 45, 51].

### Sex differences

Variability in FAox owing to sex exist due to the inherent hormonal differences specific to men and women [53–56]. In a comprehensive study with over 300 men and premenopausal women, the energy contribution of fat was significantly higher in women vs. men at all exercise intensities measured ranging from 41-61% VO_2max_ [2]. Studies have consistently shown that premenopausal women have a significantly greater ability to oxidize fat during exercise [2, 57, 58].

The sex differences in fat oxidation [58, 59] during exercise is attributed to the increased circulation of estrogens [53, 54, 60]. Evidence suggests that estrogen directly stimulates AMPK [29] and PGC-1α activity [60], which is thought to increase the downstream FAox transport protein CD36 and beta-oxidative protein HAD [30]. Additionally, beta-oxidative proteins that oxidize LCFA oxidation have been shown to be regulated in part by estrogen [54, 60]. The result of increased beta-oxidative proteins is directly related to increased FAox potential [29, 54]. Interestingly, when men were supplemented with estrogen, increases in FAox were observed along with increased cellular expression of beta-ox proteins within eight days of supplementation [60].

Circulating estrogen is naturally higher for premenopausal women compared to men. Additionally, fluctuation in estrogen levels is inherent throughout the menstrual cycle [53, 59]. Estrogens are generally higher during the follicular phase of the menstrual cycle compared to the luteal phase [29]. Paradoxically, elevated estrogens during the follicular phase do not affect FAox when compared to the luteal phase [29, 53]. Nevertheless, elevations in endogenous circulating estrogens inherent to premenopausal women increase the expression of cellular proteins responsible for increased FA transport and oxidation compared to men.

### Nutrition

Cellular protein expression and the corresponding endogenous vs. systematic substrate oxidation vary according to dietary macronutrient intake [19, 35, 61]. It has been recently shown that high fat diets promote FAox and have performance enhancement capabilities [3, 60]. However, definitive conclusions regarding pre-exercise macronutrient dominant diets and exercise performance improvements are contingent on specific exercise applications [62] that are directed by exercise duration and intensity [63–65].

Diets that have higher proportions of a specific macronutrient (e.g. fat/CHO) have shown an increased ability to oxidize the primary macronutrient consumed [66–68]. Furthermore, endogenous substrate concentrations increase after acclimating to high fat/high CHO diets [65, 68, 69]. High fat diets increase IMTG concentrations while decreasing glycogen levels within muscle [17, 35]. Alternatively, high CHO diet conditions increase glycogen concentrations while IMTGs decrease [17]. After acclimation, during exercise the body favors oxidation of specific substrates [65, 67] based on long-term (>48 h) cellular adaptation in accordance to macronutrient consumption [3, 35, 69]. However, post-exercise predominant macronutrient (CHO) consumption has been shown to influence cellular protein expression in as little as 2 hrs [69]. The plasticity of cellular changes relevant to chronic adaptation are compromised when macronutrient content is altered [65, 67].

Macronutrient proportion and timing has been shown to have effects on cellular adaptation [32] as well as the physiological response to exercise [70–72]. High fat diets increase beta-ox potential at rest [66] and during exercise [34], however, the limitations of high fat diets (including short term adaptation (5dys)) reside with high intensity exercise [70, 72, 73]. High intensity exercise (>75% VO_2max_) eclipses the FAox oxidative potential relying on fast glycolysis, or more specifically PDH to produce CHO-derived acetyl-CoA [24] for ATP re-synthesis [35, 67]. Pyruvate dehydrogenase is the enzyme responsible for oxidizing pyruvate as the final substrate of the glycolytic pathway. The deleterious cellular adaptation of reduced PDH activity due to high fat diets has been found to compromise high intensity exercise performance potential [35, 63, 67].

High fat diets (>68% total daily calorie intake) have had positive effects on lowering RER values [64, 71, 72] during moderate intensity exercise (~64–70% VO_2max_), and for prolonged exercise durations (~3 hrs) [34] indicating an increase in FAox. Adapting the body to high fat diets allows the body to increase IMTG storage as well as increase FAox [21, 35]. Contrariwise, PDH activity and therefore CHO oxidation was shown to be compromised [35, 67] along with power output at exercise intensities ≥70% VO2max [73]. However, crossover diet applications where the body was adapted to a high fat diet prior to short term high CHO loading (36-72 h) was shown to maintain IMTG stores [65] while increasing glycogen stores [72], partially restore glycolytic enzymes [35], as well as partially restore CHOox [67]. Increasing MFO (g/min) and the exercise intensity that MFO occurs (% VO2max) [34] is ideal for long duration exercise performance. However, further research on the initial compromised cellular PDH activity after fat adaptation, and the capacity to restore glycolytic potential after short term CHO adaptation is warranted for prolonged intermittent high intensity exercise (≥70% VO2max) applications.

Alternating pre-exercise macronutrient specificity has the potential to be effective in accommodating the stress of sustained high intensity exercise due to both ideal cellular protein expression, and adequate storage of IMTG and muscle glycogen. The adaptation to high fat diets (>50% Fat of total K/cal) [19] has been shown to reduce PDH activity [35] by 59% at rest and 29% at moderate intensity exercise (70% VO_2max_) [67]. The reduction in PDH activity due to high fat diets is a limiting factor to the necessary CHO oxidation at high intensity exercise despite adequate endogenous energy stores. However, five days of fat adaptation (~67% of total energy intake) with a 24 hr short-term CHO loading period (compared with high CHO diet ~70% total energy intake) maintains IMTG concentrations and partially restores PDH activity (71% of high CHO diet) while maintaining 80% HSL activity [67]. Additionally, no differences were observed in time trial performance (~10 min at 90% VO_2peak_) between the high CHO and high fat/short term CHO diet adaptation [67]. Maintaining the ability to store and oxidize fat after acclimating to a high fat diet while restoring the ability to oxidize CHO with short-term CHO loading is an ideal physiological state for endurance exercise performance. Furthermore, glycogenolysis is elevated during exercise after CHO loading [67] indicating an increase in both glycogen storage as well as an increased ability to produce/maintain CHO availability during intense exercise [71].

Current research asserts that high fat diets favorably enhance FAox at both rest and during exercise [3, 74]. However, exercise intensity dictates substrate utilization regardless of dietary influence, training status, and exercise duration. Because of this, high fat diets are sometimes encouraged during preparatory off-season training when training volumes are high and exercise intensities are low to moderate [74]. However, during sustained high intensity exercise (>70% VO_2max_) which is common during competition, CHO is the primary substrate relied upon despite short and long term fat acclimation [71, 75]. More research into the short-term macronutrient manipulation effect on endogenous substrate concentrations, plasticity of cellular expression, and preferential substrate oxidation are necessary to ascertain if there is benefit on exercise performance outcomes.

## Conclusion

In summary, FAox is contingent on many factors which can modify cellular expression in a short amount of time. Macronutrient availability, training status, sex, exercise intensity, and duration all influence cellular adaptation, systematic FA transport, and FAox. Exercise intensity dominates substrate oxidation acutely, regardless of training status and/or nutritional influence. Additionally, more investigation into the ideal nutritional timing and content that will favorably influence the physiological adaptations of FAox during endurance exercise is warranted. Nonetheless, exercise prescriptions and dietary recommendations need to take into account specific exercise goals (duration, intensity, sport specific) to facilitate a training plan that will elicit the ideal substrate oxidation adaptations relevant to improve sport performance.

## Abbreviations

~: Approximately
<: Less than
>: Greater than
±: Plus or minus
≤: Less than or equal to
≥: Greater than or equal to
AMPK: 5′ adenosine monophosphate-activated protein kinase
ATGL: Adipose triglyceride lipase
ATP: Adenosine triphosphate
Beta-ox: Beta oxidation
BF%: Body fat percentage
CD36: Cluster of differentiation 36
CHO: Carbohydrate
CPT-1: Carnitine palmitoyltransferase-1
CPT-II: Carnitine palmitoyltransferase-II
DAG: Diacylglycerol
FA: Fatty acid
FAox: Fatty acid oxidation
FFM: Fat free mass
FM: Fat mass
g/min: Grams per minute
HAD: β-hydroxy acyl-CoA dehydrogenase
HR: Heart rate
HSL: Hormone sensitive lipase
IMTG: Intramuscular triacylglyceride
kg: Kilogram
LCFA: Long chain fatty acids
LPL: Lipoprotein lipase
MCFA: Medium chain fatty acid
MFO: Maximal fat oxidation
PDH: Pyruvate dehydrogenase
PGC-1α: Peroxisome proliferator-activated receptor gamma co-activator
Ra: Rate of appearance
RER: Respiratory exchange ratio
TAG: Triacylglycerol
TCA cycle: Tricarboxylic acid cycle
VO_2_: Volume of oxygen consumed
VO_2max_: Maximal oxygen consumption
yrs.: Years

## References

[1] Achten J, Jeukendrup A (2004). Optimizing fat oxidation through exercise and diet. Nutrition.

[2] Venables M, Achten J, Jeukendrup AE (2005). Determinants of fat oxidation during exercise in healthy men and women: a cross-sectional study. J Appl Phys.

[3] Volek JS, Noakes T, Phinney SD (2015). Rethinking fat as a fuel of endurance exercise. Eur J Sport Sci.

[4] Brooks GA, Mercier J (1994). Balance of carbohydrate and lipid utilization during exercise: the "crossover" concept. J Appl Phys.

[5] Achten J, Gleeson M, Jeukendrup AE (2002). Determination of the exercise intensity that elicits maximal fat oxidation. Med Sci Sports Exerc.

[6] Valizadeh A, Khosravi A, Azmoon H (2011). Fat oxidation rate during and after three exercise intensities in non-athlete young men. World Appl Sci J.

[7] Randell RK, Rollo I, Roberts TJ, Dalrymple KJ, Jekendrup AE, Carter JM (2017). Maximal fat oxidation rates in an athletic population. Med Sci Sports Exerc.

[8] Ogasawara J, Izawa T, Sakurai T, Sakurai T, Shirato K, Ishibashi Y, Ishida H, Ohno H, Kizaki T. The molecular mechanism underlying continuous exercise training-induced adaptive changes of lipolysis in white adipose cells. J Obesity. 2015; 10.1155/2015/473430. 10.1155/2015/473430 PMC4444571 26075089

[9] Watt M, Spriet LL (2010). Triacylglycerol lipases and metabolic control: implications for health and disease. Am J of Physol. Endocrinol Metab.

[10] Zechner R, Kienesberger PC, Haemmerle G, Zimmermann R, Lass A (2009). Adipose triglyceride lipase and the lipolytic catabolism of cellular fat stores. J Lipid Res.

[11] van Loon L, Greenhaff PL, Constantin-Teodosiu D, Wagenmakers AJ (2001). The effects of increasing exercise intensity on muscle fuel utilisation in humans. J Physiol.

[12] Tank A, Wong D (2015). Peripheral and central effects of circulating catecholamines. Compr Physol.

[13] van Hall G. THe physiological regulation of skeletal muscle fatty acid supply and oxidation during moderate-intensity exercise. Sports Med. 2015;Suppl 1:S23-S32. 10.1007/s40279-015-0394-8 PMC4672010 26553490

[14] Zouhal H, Jacob C, Delamarche P, Grata-Delamarche A (2008). Catecholamines and the effects of exercise, training and gender. Sports Med.

[15] Horowitz J, Klein S (2000). Lipid metabolism during endurance exercise. Am J Clin Nutr.

[16] Frayn K (2010). Fat as fuel: emerging understanding of the adipose tissue-skeletal muscle axis. Acta Physiol.

[17] Spriet LL (2014). New insights into the interaction of carbohydrate and fat metabolism during exercise. Sports Med.

[18] Kiens B (2006). Skeletal muscle lipid metabolism in exercise and insulin resistance. Physol Rev.

[19] Shaw C, Clark J, Wagenmakers A (2010). The effect of exercise and nutrition on intramuscular fat metabolism and insulin sensitivity. Annu Rev Nutr.

[20] Moro C, Bajpeyi S, Smith SR (2007). Determinants of intramyocellular triglyceride turnover: implications for insulin sensitivity. Am J Physiol Endocrinol Metab.

[21] Wong H, Schotz MC (2002). The lipase gene family. J Lipid Res.

[22] van Loon L, Greenhaff P, Constantin-Teodosiu D, Saris W, Wagenmakers A (2004). Use of intramuscular triacylgylcerol as a substrate source during exercise in humans. J Appl Physiol.

[23] Watt M, Heigenhauser G, Spriet LL (2002). Intramuscular triacylgylerol utilization in human skeletal muscle during exericse: is there a controversy?. J Appl Physiol.

[24] Jeppesen J, Keins B (2012). Regulation and limitations to fatty acid oxidation during exercise. J Phys.

[25] Yoshida Y, Jain SS, McFarlan JT, Snook LA, Chabowski A, Bonen A (2013). Exercise- and training-induced upregulation of skeletal muscle fatty acid oxidation are not solely dependent on mitochondrial machinery and biogenesis. J Physiol.

[26] Schenk S, Horowitz JF (2006). Coimmunoprecipitation of FAT/CD36 and CPT I in skeletal muscle increases proportionally with fat oxidation after endurance exercise training. Am J Physiol Endocrinol Metab.

[27] Klien S, Coyle E, Wolfe R (1994). Fat metabolism during low-intensity exercise in endurance-trained and untrained men. Am J Phys.

[28] Lundsgaard A, Kiens B (2014). Gender differences in skeletal muscle substrate metabolism-molecular mechanisms and insulin sensitivity. Front Endocrinol.

[29] Oosthuyse T, Bosch A (2010). The effect of the menstual cycle on exercise metabolism. Sports Med.

[30] Kiens B, Roepstorff C, Glatz J, Bonen A, Schjerling P, Knudsen J, Nielsen J (2004). Lipid-binding proteins and lipoprotein lipase activity in human skeletal muscle: influence of physical activity and gender. J Appl Physiol.

[31] DeLany J, Windhauser M, Champagne C, Bray G (2000). Differential oxidation of individual dietary fatty acids in humans. Am J Clin Nutr.

[32] Misell L, Lagomarcino N, Shuster V, Kern M (2001). Chronic medium-chain triacylglycerol consumption and endurance performance in trained runners. J Sports Med Phys Fit.

[33] Jeukendrup A, Aldred S (2004). Fat supplementation, health, and endurance performance. Nutr.

[34] Volek J, Freidenreich D, Saenz C, Kunces L, Creighton B, Bartley, Davitt P, Munoz C, Anderson J, Maresh C, Lee E, Schuenke M, Aerni G, Kramer W, Phinney S (2016). Metabolic characteristics of keto-adapted ultra-endurance runners. Metab.

[35] Yeo W, Carey A, Burke L, Spriet LL, Hawley J (2011). Fat adaptation in well-trained athletes: effects on cell metabolism. Appl Physiol Nutr Metab.

[36] Jeukendrup AE (1998). Fat metabolism during exercise: a review. Part III: effects of nutritional interventions. Int J Sports Med.

[37] Calvani M, Reda E, Arrigoni-Martelli E (2000). Regluation by carnitine of myocardial fatty acid and carbohydrate metabolism under normal and pathological conditions. Basic Res Cardiol.

[38] Stephens F, Constantin-Teodosiu D, Greenhaff P (2007). New insights concerning the role of carnitine in the regulaiton of fuel metabolism in skeletal muscle. J Physiol.

[39] Lima-Silva A, Bertuzzi R, Pires F, Gagliardi J, Barros R, Hammond J, Kiss M (2010). Relationship between training status and maximal fat oxidation. J Sports Sci Med..

[40] Scharhag-Rosenberger FM, Meyer T, Walitzek S, Kindermann W. Effects of one year aerobic endurance training on resting metabolic rate and exercise fat oxidation in previously untrained men and women. Metabolic endurance training adaptations. Int J Sports Med. 2010;31:498–504. 10.1055/s-0030-1249621 20432193

[41] Bircher S, Knechtle B (2004). Relationship between fat oxidation and lactate threshold in athletes and obese women and men. J Sports Sci Med.

[42] Nordby P, Saltin B, Helge JW (2006). Whole-body fat oxidation determined by graded exercise and indirect calorimetry: a role for muscle oxidative capacity?. Scand J Med Sci Sports.

[43] Lanzi S, Codecasa F, Cornacchia M, Maestrini S, Slvadori A, Brunani A, Malatesta D. Fat oxidation, hormonal and plasma metabolite kinetics during a submaximal incremental test in lean and obese adults. PLoS One. 2014;9(2) 10.1371/journal.pone.0088707. 10.1371/journal.pone.0088707 PMC3921204 24523934

[44] Stisen A, Stougaard O, Langfort J, Helge J, Sahlin K, Madsen K (2006). Maximal fat oxidation rates in endurance trained and untrained women. Eur J Appl Physiol.

[45] Watt M, Heigenhauser G, Dyck D, Spriet LL (2002). Intramuscular triacylglycerol, glycogen, and acetyl group metabolism during 4 h of moderate exercise in man. J Physiol.

[46] Mora-Rodriguez R, Hodgkinson BJ, Byerley LO, Coyle EF (2001). Effects of -adrenergic receptor stimulation and blockade on substrate metabolism during submaximal exercise. Am J Physol. Endocrinol Metab.

[47] Martin W (1996). Effects of acute and chronic exercise on fat metabolism. Exerc Sport Sci Revs.

[48] Romijn J, Coyle E, Sidossis L, Gastaldelli A, Horowitz J, Endert E, Wolfe R (1993). Regulation of endogenous fat and carbohydrate metabolism in relation to exercise intensity and duration. Am J phys. Endocrinol Metab.

[49] Bergomaster K, Howarth KR, Phillips SM, Rakobowchuk M, MacDonald MJ, McGee SL, Gibala MJ (2008). Similar metabolic adaptations during exercise after low volume sprint interval and traditional endurance training in humans. J Phys.

[50] Astorino T (2000). Is the ventilatory threshold coincident with maximal fat oxidation during submaximal exercise in women?. J Sports Med Phys Fitness.

[51] Turcotte L, Richeter E, Kiens B (1992). Increased plasma FFA uptake and oxidation during prolonged exericse in trained vs. untrained humans. Am J Physiol Endocrinol Metab.

[52] Martin W (1997). Effect of endurance training on fatty acid metabolism during whole body exercise. Med Sci Sports Exerc.

[53] Isacco L, Duché P, Buisseau N (2012). Influence of hormonal status on substrate utilization at rest and during exercise in the female population. Sports Med.

[54] Maher A, Akhtar M, Vockley J, Tarnopolosky M. Women have higher protein content of beta oxidation enzymes in skeletal muscle than men. PLoS One. 2010;5(8) 10.1371/journal.pone.0012025. 10.1371/journal.pone.0012025 PMC2917369 20700461

[55] Tarnopolosky M (2008). Sex differences in exercise metabolism and the role of 17-beta estradiol. Med Sci Sports Exerc.

[56] Varmlamov O, Bethea CL, Roberts CT (2015). Sex-specific differences in lipid and glucose metabolism. Front Endocrinol.

[57] Dasilva SG, Guidetti L, Buzzachera CF, Elsangedy HM, Krinski K, De Campos W, Goss FL, Baldari C (2011). Gender-based differences in substrate use during exercise at a self-selected pace. J Strength Cond Res.

[58] Carter S, Rennie C, Tarnopolosky M (2001). Substrate utilization during endurance exercise in men and women after endurance training. Am J Endocrinoly Metab.

[59] Lebrun C (1993). Effect of the different phases of the menstrual cycle and oral contraceptives on athletic performance. Sports Med.

[60] Maher A, Akhtar M, Tarnopolsky M (2010). Men supplemented with 17b-estradiol increased b-oxidation capacity in skeletal muscle. Physiol Genomics.

[61] Fletcher G, Eves FF, Glover EI, Robinson SL, Vernooij CA, Thompson JL, Wallis GA (2017). Dietary intake is independently associated with the maximal capacity for fat oxidation during exercise. Am J Clin Nutr.

[62] Phinney S (2004). Ketogenic diets and physical performance. Nutr Metab.

[63] Burke L (2015). Re-examining high-fat diets for sports perfomance: did we call the 'nail in the coffin' too soon?. Sports Med.

[64] Hawley J, Leckey J (2015). Carbohydrate dependence during prolonged, intense endurance exercise. Sports Med.

[65] Ochiai M, Matsuo T (2009). Effects of short-term dietary change from high-carbohydrate diet to high-fat diet on storage, utilization, and fatty acid composition of rat muscle triglyceride during swimming exercise. J Clin Biochem Nutr.

[66] Miles-Chan J, Dulloo AG, Schutz Y (2015). Fasting substrate oxidation at rest assessed by indirect calorimetry: is prior dietary macronutrient level and composition a confounder?. Int J Obes.

[67] Stellingwerff T, Spriet LL, Watt M, Kimber N, Hargreaves M, Hawley J, Burkey L. Decreased PDH activiation and glycogenolysis during exercise following fat adaptation with carbohydrate resortation. Am J Endocrinol Metab. 2006;290:E380–8. 10.1152/ajpendo.00268.2005 16188909

[68] Vogt M, Puntschart A, Haowald J, Mueller B, Mannahart C, Gfeller-Teuscher L, Mullis P, Hoppeler H (2003). Effects of dietary fat on muscle substrates, metabolism, and performance in athletes. Med Sci Sports Exerc.

[69] Pilegaard H, Keller C, Seensberg A, Helge J, Pedersen B, Saltin B, Neufer D (2002). Influence of pre-exercise muscle glycogen content on exercise-induced transcriptional regulation of metabolic genes. J Physiol.

[70] Burke L, Hawley J, Angus D, Cox G, Clark S, Cummings N, Desbrow B, Hargreaves M (2002). Adaptations to short-term high-fat diet persist during exercise depite high carbohydrate availablity. Med Sci Sports Exerc.

[71] Webster C, Noakes T, Chacko S, Swart J, Kohn T, Smith J. Gluconeogenesis during endurance exercise in cyclists habituated to a long-term low carbohydrate high fat diet. J Physiol. 2016. 10.1113/JP271934. 10.1113/JP271934 PMC4967730 26918583

[72] Zehnder M, Christ E, Ith M, Acheson KJ, Pouteau E, Kreis R, Trepp R, Diem P, Boesch C, Décombaz J (2006). Intramyocellular lipid stores increase markedly in athletes after 1.5 days lipid supplementation and are utilized during exercise in proportion to their content. Eur J Appl Physiol.

[73] Havemann L, West S, Goedecke J, Macdonald L, St. Clair Gibson A, Noakes T, Lambert E (2006). Fat adaptation followed by carbohydrate loading compromises high-intensity sprint performance. J Appl Physiol.

[74] Zajac P, Poprzecki S, Maszczyk A, Czuba M, Michalczyk M, Zydek G (2014). The effects of a ketogenic diet on exercise metabolism and physical performance in off-road cyclists. Nutrients.

[75] Leckey J, Burke J, Morton J, Hawley J. Altering fatty acid availability does not impair prolonged, continuous running to fatigue: evidence for carbohydrate dependence. J of Appl Physiol. 2016;120(3):107–13. 10.1152/japplphysiol.00855.2015 26586912

